# Supplementary material for: Polyketide synthase gene clusters in the ericoid mycorrhizal fungus Oidiodendron maius: genome-wide prediction, expression profiling, and functional characterization of knockout mutants
Source: Front Plant Sci. 2026 Sep 16;17:1924430. doi: 10.3389/fpls.2026.1924430 (PMC13623903; doi:10.3389/fpls.2026.1924430)
Supplement: Supplementary file 1 [file SupplementaryFile1.zip › Supplementary Materials/Supplementary Folder 1.zip!/Oidma1_AssemblyScaffolds_Repeatmasked/knownclusterblast/cluster54/jgi.p_Oidma1_62072_mibig_hits.html]

| MIBiG Protein | Description | MIBiG Cluster | MiBiG Product | % ID | % Coverage | BLAST Score | E-value |
| --- | --- | --- | --- | --- | --- | --- | --- |
| ACD39771.1 | non-heme\_halogenase | BGC0000134 | Radicicol\_biosynthetic\_gene\_cluster | 67 | 102.5 | 738 | 5.1e-213 |
| ADI24948.1 | GsfI | BGC0000070 | Griseofulvin\_biosynthetic\_gene\_cluster | 65 | 99.8076923077 | 700 | 1.2e-201 |
| AGO59046.1 | PtaM | BGC0000121 | Pestheic\_acid\_biosynthetic\_gene\_cluster | 51 | 100.0 | 551 | 8.9e-157 |
| BAE56588.1 |  | BGC0001123 | Aspirochlorine\_biosynthetic\_gene\_cluster | 52 | 101.346153846 | 544 | 1.4e-154 |
| EAQ86388.1 | hypothetical\_protein | BGC0001405 | Chaetoviridin\_/\_chaetomugilin\_biosynthetic\_gene\_cluster | 52 | 101.730769231 | 535 | 8.6e-152 |
| AEM76785.1 | putative\_halogenase | BGC0001401 | Melleolides\_biosynthetic\_gene\_cluster | 45 | 102.5 | 435 | 7.1e-122 |
| ALT31850.1 | ArmH3 | BGC0001401\_c3 | Melleolides\_biosynthetic\_gene\_cluster | 49 | 95.7692307692 | 429 | 5.1e-120 |
| AEM76786.1 | putative\_halogenase | BGC0001401\_c2 | Melleolides\_biosynthetic\_gene\_cluster | 46 | 89.0384615385 | 413 | 2.9e-115 |
| ALT31851.1 | ArmH4 | BGC0001401\_c4 | Melleolides\_biosynthetic\_gene\_cluster | 50 | 86.3461538462 | 404 | 1.7e-112 |
| ALT31852.1 | ArmH5 | BGC0001401\_c5 | Melleolides\_biosynthetic\_gene\_cluster | 41 | 103.653846154 | 396 | 3.6e-110 |
| ABM21576.1 | CrpH | BGC0000975 | Cryptophycin\_biosynthetic\_gene\_cluster | 40 | 74.2307692308 | 277 | 3.2e-74 |
| AMK51270.1 | Hex13 | BGC0001376 | Hexaricin\_biosynthetic\_gene\_cluster | 39 | 74.4230769231 | 271 | 2.3e-72 |
| ADE22292.1 | putative\_non-heme\_halogenase | BGC0000279 | Xantholipin\_biosynthetic\_gene\_cluster | 37 | 82.6923076923 | 268 | 2e-71 |
| CAJ46693.1 | tryptophan\_halogenase | BGC0000969 | Chondramide\_biosynthetic\_gene\_cluster | 37 | 75.5769230769 | 255 | 1.7e-67 |
| CAM34371.1 | putative\_non-heme\_halogenase | BGC0000242 | Lysolipin\_biosynthetic\_gene\_cluster | 36 | 81.3461538462 | 250 | 3.2e-66 |
| ACU71639.1 | tryptophan\_halogenase | BGC0001154 | Cacibiocin\_biosynthetic\_gene\_cluster | 34 | 71.5384615385 | 215 | 1.2e-55 |
| WP\_052390547.1 | halogenase | BGC0001308 | Simocyclinone\_D9\_/\_simocyclinone\_D10\_/\_simocyclinone\_D11 | 33 | 71.5384615385 | 205 | 1.6e-52 |
| AEU17894.1 | putative\_halogenase | BGC0001072 | Simocyclinone\_biosynthetic\_gene\_cluster | 32 | 73.0769230769 | 201 | 1.7e-51 |
| ADU85999.1 | putative\_tryptophan\_halogenase | BGC0000165 | Tiacumicin\_B\_biosynthetic\_gene\_cluster | 32 | 71.5384615385 | 196 | 5.5e-50 |
| AGC09515.1 | FAD\_dependent\_halogenase | BGC0001183 | Lobophorin\_biosynthetic\_gene\_cluster | 34 | 73.2692307692 | 190 | 4e-48 |
| CAE53362.1 | halogenase | BGC0000440 | Teicoplanin\_biosynthetic\_gene\_cluster | 33 | 75.7692307692 | 189 | 8.8e-48 |
| CAG15020.1 | halogenase | BGC0000441 | Teicoplanin\_biosynthetic\_gene\_cluster | 33 | 75.7692307692 | 189 | 8.8e-48 |
| AGS77325.1 | Halogenase | BGC0001178 | UK-68,597\_biosynthetic\_gene\_cluster | 33 | 72.6923076923 | 188 | 2e-47 |
| AAK81830.1 | non-heme\_halogenase | BGC0000326 | Complestatin\_biosynthetic\_gene\_cluster | 33 | 72.8846153846 | 187 | 4.4e-47 |
| AEI58871.1 | halogenase | BGC0000455 | Vancomycin\_biosynthetic\_gene\_cluster | 32 | 75.7692307692 | 186 | 7.5e-47 |
| ctg1\_orf12 |  | BGC0001366 | BE-24566B\_/\_zunyimycin\_A\_biosynthetic\_gene\_cluster | 34 | 75.7692307692 | 183 | 4.8e-46 |
| CAA76550.1 | halogenase | BGC0000311 | Balhimycin\_biosynthetic\_gene\_cluster | 32 | 75.9615384615 | 183 | 8.3e-46 |
| AAM80532.1 | StaI | BGC0000290 | A47934\_biosynthetic\_gene\_cluster | 33 | 77.1153846154 | 181 | 2.4e-45 |
| CAA11780.1 | PCZA361.26 | BGC0000322\_c2 | Chloroeremomycin\_biosynthetic\_gene\_cluster | 32 | 75.9615384615 | 180 | 5.4e-45 |
| CAD91205.1 | halogenase | BGC0000289 | A40926\_biosynthetic\_gene\_cluster | 31 | 73.6538461538 | 179 | 9.1e-45 |
| AFV52210.1 | aryl-ACP\_halogenase | BGC0000081 | Kedarcidin\_biosynthetic\_gene\_cluster | 33 | 72.5 | 178 | 2.7e-44 |
| ALD82533.1 | halogenase | BGC0001212 | Nannocystin\_biosynthetic\_gene\_cluster | 34 | 68.6538461538 | 176 | 7.7e-44 |
| ABY66006.1 | halogenase | BGC0001008 | Maduropeptin\_biosynthetic\_gene\_cluster | 33 | 69.2307692308 | 174 | 3.8e-43 |
| AAL06656.1 | alkylhalidase | BGC0000965 | C-1027\_biosynthetic\_gene\_cluster | 32 | 71.3461538462 | 172 | 1.5e-42 |
| ABD65950.1 | halogenase | BGC0000341 | Enduracidin\_biosynthetic\_gene\_cluster | 32 | 72.6923076923 | 171 | 2.5e-42 |
| ALU98528.1 | tryptophan\_halogenase | BGC0001397 | Lidamycin\_biosynthetic\_gene\_cluster | 32 | 69.8076923077 | 171 | 3.3e-42 |
| AAN65237.1 | putative\_halogenase | BGC0000832 | Clorobiocin\_biosynthetic\_gene\_cluster | 33 | 65.9615384615 | 168 | 2.1e-41 |
| AAM54090.1 | halogenase | BGC0000020 | Ansamitocin\_biosynthetic\_gene\_cluster | 32 | 74.4230769231 | 162 | 1.2e-39 |
| ABS50488.1 | NapH2 | BGC0000652 | Napyradiomycin\_biosynthetic\_gene\_cluster | 31 | 64.0384615385 | 158 | 1.7e-38 |
| ABS50460.1 | NapH2 | BGC0001079 | Napyradiomycin\_biosynthetic\_gene\_cluster | 31 | 64.0384615385 | 158 | 1.7e-38 |
| AGS77327.1 | Halogenase | BGC0001178 | UK-68,597\_biosynthetic\_gene\_cluster | 28 | 93.8461538462 | 158 | 2.8e-38 |
| ADM46362.1 | halogenase | BGC0000106 | Naphthomycin\_biosynthetic\_gene\_cluster | 30 | 74.0384615385 | 156 | 6.3e-38 |
| AAM80530.1 | StaK | BGC0000290 | A47934\_biosynthetic\_gene\_cluster | 32 | 69.2307692308 | 155 | 2.4e-37 |
| CAQ43074.1 | halogenase | BGC0000970 | Chondrochloren\_biosynthetic\_gene\_cluster | 29 | 74.2307692308 | 153 | 9.2e-37 |
| AAM70353.1 | CalO3 | BGC0000033 | Calicheamicin\_biosynthetic\_gene\_cluster | 31 | 66.5384615385 | 143 | 9.5e-34 |
| ESP90845.1 | Dehydrogenases\_(flavoprotein) | BGC0000891 | Bromophenols\_/\_bromopyrroles\_biosynthetic\_gene\_cluster | 27 | 68.4615384615 | 126 | 7e-29 |
| AHA34035.1 | Bmp2 | BGC0000890 | Bromophenols\_/\_bromopyrroles\_biosynthetic\_gene\_cluster | 28 | 67.5 | 124 | 2.7e-28 |
| AGC24269.1 | PrlO | BGC0001038 | Pyralomicin\_biosynthetic\_gene\_cluster | 25 | 72.1153846154 | 82 | 2e-15 |
| BAD38872.1 | putative\_halogenase | BGC0000111 | Neocarzilin\_biosynthetic\_gene\_cluster | 26 | 72.6923076923 | 80 | 7.5e-15 |
| AKQ20698.1 | monooxygenase | BGC0001337 | Lazarimide\_biosynthetic\_gene\_cluster | 26 | 69.0384615385 | 58 | 3.1e-08 |
